# Supplementary material for: Profiling the Abiotic Stress Responsive microRNA Landscape of Arabidopsis thaliana
Source: Plants (Basel). 2019 Mar 10;8(3):58. doi: 10.3390/plants8030058 (PMC6473545; doi:10.3390/plants8030058)
Supplement: Supplementary file 1 [file plants-08-00058-s001.pdf]

Profiling the Abiotic Stress Responsive microRNA Landscape of *Arabidopsis thaliana*

Joseph L Pegler, Jackson MJ Oultram, Christopher PL Grof and Andrew L Eamens *

Centre for Plant Science, School of Environmental and Life Sciences, Faculty of Science, University of Newcastle, Callaghan 2308, Australia

* Correspondence: andy.eamens@newcastle.edu.au; Tel.: +61-249-217-784

Supplemental Table S1: Raw miRNA reads and the Log2 fold change in abundance of each *Arabidopsis thaliana* miRNA sRNA detected via high throughput sRNA sequencing.

Using the Qiagen CLC Genomics Workbench (11) software, the sequencing adapter sequences were removed prior to performing sequence quality trimming to remove any sRNA reads that were either shorter than 15 nucleotides (nts), or longer than 35 nts in length. Additionally, parameters within the CLC Genomic Workbench were applied to remove any ambiguous nucleotides at either the 5' or 3' terminus of each sequencing read (i.e., the removal of any 'N' nucleotides on sequence ends), or to 'trim' low quality sequences using a modified '*Mott trimming*' algorithm. This approach allowed for the determination of the (1) total library size (high quality reads only) for each treatment (control, heat, drought and salt stress), and (2) the raw read count for each *Arabidopsis thaliana* miRNA sRNA detected. The total number of miRNA sRNA raw reads for each library was subsequently determined via summing the raw read counts of all detected miRNA sRNAs together per library in Microsoft® Excel 2016. Microsoft® Excel 2016 was also used to normalize miRNA abundance per library for interlibrary comparisons. Post miRNA abundance normalization for each library, the Log2 fold change in miRNA abundance per stress treatment was calculated via the division of the normalized stress library miRNA read count by the normalized non-stressed control library miRNA read count. Specifically, and using the heat stress library as an example, the Log2 fold change for each miRNA sRNA was determined using the Log2 function in Microsoft® Excel, based on the following equation: (miRNA raw reads (heat) / total library size (heat)) // miRNA raw reads (control) / total library size (control)). The calculated Log2 fold change for each miRNA sRNA was subsequently used to construct Figure 2A.

Treatment	Control	Heat		Drought		Salt	
**miRNA**	**Raw reads**	**Raw reads**	**Log2 FC**	**Raw reads**	**Log2 FC**	**Raw reads**	**Log2 FC**
**miR156a**	4901	4496	0.102	17203	1.839	8912	0.951
**miR156b**	3224	4990	0.856	13996	2.145	8087	1.415
**miR156c**	3939	4160	0.305	15082	1.964	7661	1.048
**miR156d**	3060	3399	0.378	11644	1.955	6657	1.210
**miR156e**	1757	1725	0.200	8646	2.326	3752	1.183
**miR156f**	1855	1727	0.123	8812	2.275	3762	1.108
**miR156g**	52	100	1.170	219	2.102	86	0.814
**miR156h**	8	45	2.718	16	1.027	6	-0.327
**miR156j**	7	5	-0.259	35	2.349	8	0.281
**miR157a**	4512	6652	0.786	25207	2.509	9606	1.179
**miR157b**	4513	6643	0.784	25214	2.509	9604	1.178
**miR157c**	1814	3447	1.152	8181	2.200	4911	1.525
**miR157d**	37	68	1.104	179	2.302	112	1.686

**miR158a**	93924	111812	0.478	271986	1.561	185555	1.071
**miR158b**	873	8904	3.577	2830	1.724	2519	1.617
**miR159a**	25272	41137	0.929	95303	1.942	44185	0.894
**miR159b**	28317	42855	0.824	85495	1.622	44948	0.755
**miR159c**	2292	4746	1.276	11472	2.351	4839	1.166
**miR160a**	204	438	1.329	710	1.827	351	0.871
**miR160b**	234	380	0.926	646	1.492	267	0.279
**miR160c**	401	1125	1.714	1339	1.767	1325	1.813
**miR161**	92819	85868	0.114	153129	0.750	117690	0.431
**miR162a**	8042	6261	-0.135	12887	0.708	10759	0.508
**miR162b**	8248	7003	-0.010	13248	0.711	10919	0.493
**miR163**	1569	1256	-0.095	2573	0.741	619	-1.254
**miR164a**	94	460	2.517	364	1.981	193	1.126
**miR164b**	677	1498	1.372	752	0.179	341	-0.901
**miR164c**	175	473	1.661	578	1.751	337	1.034
**miR165a**	3567	5693	0.901	8410	1.265	4668	0.476
**miR165b**	4401	5898	0.649	9193	1.090	5033	0.282
**miR166a**	4806	5665	0.463	10678	1.179	6513	0.527
**miR166b**	3882	4509	0.442	8799	1.208	5290	0.535
**miR166c**	3167	3336	0.301	6779	1.125	3761	0.336
**miR166d**	3101	3266	0.301	6670	1.132	3687	0.338
**miR166e**	4640	4929	0.313	9798	1.106	5461	0.323
**miR166f**	4546	4681	0.268	9353	1.068	5298	0.309
**miR166g**	4539	4650	0.261	9325	1.066	5287	0.308
**miR167a**	3229	5956	1.109	9467	1.579	5473	0.850
**miR167b**	2896	5285	1.094	8521	1.584	4959	0.864
**miR167c**	1326	961	-0.238	3071	1.239	1628	0.384
**miR167d**	1890	2843	0.815	5913	1.673	3631	1.030
**miR168a**	2566	5231	1.254	5921	1.234	5026	1.058
**miR168b**	1322	2397	1.085	4133	1.672	3046	1.293
**miR169a**	40	23	-0.572	51	0.378	30	-0.327
**miR169b**	33	9	-1.648	28	-0.210	11	-1.497
**miR169d**	211	13	-3.794	375	0.857	28	-2.825
**miR169e**	207	24	-2.882	293	0.529	10	-4.283
**miR169f**	263	24	-3.228	538	1.060	76	-1.703
**miR169g**	214	13	-3.815	378	0.848	23	-3.130
**miR169h**	14	2	-2.581	31	1.174	2	-2.719
**miR169i**	19	6	-1.437	49	1.394	18	0.010
**miR169j**	4	1	-1.774	12	1.612	2	-0.912
**miR169k**	4	1	-1.774	10	1.349	2	-0.912
**miR169l**	8	0	0.000	11	0.487	1	-2.912
**miR169m**	7	2	-1.581	17	1.307	0	0.000
**miR170**	256	380	0.796	481	0.937	312	0.374

**miR171a**	405	572	0.724	793	0.997	547	0.522
**miR171b**	102	205	1.233	165	0.721	82	-0.227
**miR171c**	122	162	0.635	132	0.141	99	-0.213
**MIR172a**	68	207	1.832	59	-0.177	49	-0.384
**miR172b**	91	326	2.067	93	0.059	118	0.463
**miR172c**	6	12	1.226	8	0.442	5	-0.175
**miR172d**	13	11	-0.015	15	0.234	10	-0.290
**miR172e**	24	131	2.675	48	1.027	55	1.285
**miR173**	19410	15084	-0.138	28368	0.575	18831	0.045
**miR319a**	2287	2714	0.473	3403	0.601	2090	-0.042
**miR319b**	1187	2961	1.545	2168	0.896	1630	0.546
**miR319c**	8729	2906	-1.361	11432	0.417	4893	-0.747
**miR390a**	520	839	0.916	955	0.904	725	0.568
**miR390b**	331	599	1.082	537	0.725	503	0.692
**miR391**	82	203	1.534	468	2.540	146	0.921
**miR393a**	53	172	1.925	170	1.709	75	0.589
**miR393b**	784	3644	2.443	2076	1.432	1707	1.211
**miR394a**	101	260	1.590	324	1.709	180	0.922
**miR394b**	118	280	1.473	379	1.711	205	0.885
**miR395a**	29	2217	6.483	82	1.527	521	4.255
**miR395b**	36	350	3.508	42	0.250	177	2.386
**miR395c**	36	344	3.483	41	0.215	173	2.353
**miR395d**	17	1166	6.326	39	1.225	390	4.608
**miR395e**	29	2206	6.475	79	1.473	518	4.247
**miR395f**	28	338	3.820	31	0.174	166	2.656
**miR396a**	4316	10849	1.556	12785	1.594	8549	1.074
**miR396b**	7784	19808	1.574	21346	1.483	12346	0.754
**miR397a**	7	56	3.226	11	0.679	30	2.188
**miR397b**	8	41	2.584	1	-2.973	20	1.410
**miR398a**	457	1080	1.467	717	0.677	664	0.627
**miR398b**	10077	25333	1.556	14693	0.571	17800	0.909
**miR398c**	10072	25318	1.556	14698	0.573	17763	0.907
**miR399a**	386	3263	3.306	1306	1.786	2997	3.045
**miR399b**	322	485	0.817	999	1.661	550	0.861
**miR399c**	341	529	0.860	1020	1.608	579	0.852
**miR399d**	8	140	4.356	33	2.072	58	2.946
**miR399e**	4	9	1.396	11	1.487	10	1.410
**miR399f**	25	119	2.477	56	1.191	94	1.999
**miR400**	758	213	-1.605	636	-0.226	479	-0.574
**miR401**	43	92	1.324	65	0.623	40	-0.016
**miR402**	42	205	2.513	124	1.589	84	1.088
**miR403**	7799	9768	0.551	15811	1.047	12392	0.756
**miR404**	40	55	0.686	35	-0.165	21	-0.841

**miR405a**	88	109	0.535	127	0.557	81	-0.031
**miR405b**	24	20	-0.037	34	0.530	14	-0.689
**miR405d**	11	11	0.226	25	1.212	5	-1.049
**miR406**	95	39	-1.058	64	-0.542	37	-1.272
**miR407**	115	90	-0.127	125	0.148	81	-0.417
**miR408**	2242	15808	3.044	4828	1.134	12273	2.541
**miR416**	14	17	0.506	21	0.612	8	-0.719
**miR447a**	488	666	0.675	890	0.894	649	0.500
**miR447b**	59	62	0.298	89	0.620	48	-0.209
**miR447c**	1	3	1.811	11	3.487	6	2.673
**miR472**	221	517	1.452	637	1.555	539	1.375
**miR771**	4	2	-0.774	97	4.627	14	1.896
**miR773a**	230	203	0.046	1068	2.243	802	1.890
**miR773b**	6	26	2.342	58	3.300	43	2.930
**miR775**	495	1190	1.492	1528	1.654	771	0.728
**miR778**	1	13	3.927	7	2.835	32	5.088
**miR779**	205	730	2.059	577	1.520	349	0.856
**miR780**	6	6	0.226	12	1.027	3	-0.912
**miR781a**	92	155	0.979	152	0.752	212	1.293
**miR781b**	75	160	1.319	146	0.988	167	1.243
**miR822**	714	612	0.004	1582	1.175	1822	1.440
**miR823**	160	331	1.275	376	1.260	507	1.752
**miR824**	303	1122	2.115	1093	1.878	916	1.684
**miR825**	125	109	0.029	176	0.521	92	-0.354
**miR826a**	7	8	0.419	6	-0.195	32	2.281
**miR827**	318	929	1.773	738	1.242	1338	2.161
**miR829**	168	1001	2.801	309	0.907	337	1.093
**miR831**	43	20	-0.878	74	0.811	337	3.059
**miR833a**	76	96	0.563	205	1.459	216	1.595
**miR833b**	18	16	0.056	41	1.215	51	1.591
**miR835**	86	200	1.444	225	1.415	301	1.896
**miR836**	70	41	-0.545	67	-0.036	32	-1.041
**miR837**	69	109	0.886	223	1.720	131	1.013
**miR838**	22	64	1.767	60	1.475	43	1.055
**miR839**	25	24	0.167	37	0.593	30	0.351
**miR840**	117	408	2.028	450	1.971	570	2.373
**miR841a**	243	192	-0.114	330	0.469	500	1.129
**miR841b**	238	107	-0.927	202	-0.209	214	-0.065
**miR842**	12	24	1.226	39	1.728	39	1.789
**miR843**	70	33	-0.859	113	0.718	59	-0.158
**miR844**	31	39	0.557	63	1.050	44	0.594
**miR845a**	559	333	-0.521	774	0.497	1638	1.639
**miR845b**	15	48	1.904	16	0.120	42	1.574

**miR846**	1717	2750	0.906	4303	1.353	2159	0.419
**miR847**	18	154	3.323	66	1.902	13	-0.381
**miR848**	407	370	0.089	358	-0.158	385	0.008
**miR849**	16	20	0.548	18	0.197	28	0.896
**miR850**	314	156	-0.783	521	0.758	1142	1.951
**miR851**	2	2	0.226	61	4.958	0	0.000
**miR852**	42	70	0.963	57	0.468	173	2.131
**miR853**	39	90	1.433	72	0.912	37	0.012
**miR854a**	13	16	0.526	19	0.575	45	1.880
**miR854b**	16	17	0.314	24	0.612	49	1.703
**miR854c**	20	12	-0.511	19	-0.047	43	1.193
**miR854d**	16	22	0.686	15	-0.066	41	1.446
**miR854e**	14	20	0.741	25	0.864	51	1.953
**miR855**	52	39	-0.189	63	0.304	46	-0.089
**miR857**	11	161	4.098	4	-1.432	107	3.370
**miR858a**	79	389	2.526	301	1.957	146	0.974
**miR858b**	4	44	3.686	27	2.782	13	1.789
**miR859**	5	57	3.737	17	1.793	34	2.854
**miR860**	11	20	1.089	22	1.027	28	1.436
**miR861**	67	50	-0.196	103	0.648	57	-0.145
**miR862**	25	13	-0.717	51	1.056	27	0.199
**miR863**	5829	1569	-1.667	5875	0.039	12391	1.176
**miR864**	2	48	4.811	5	1.349	4	1.088
**miR865**	197	309	0.876	315	0.705	73	-1.344
**miR866**	102	558	2.678	335	1.743	920	3.261
**miR869**	398	68	-2.323	247	-0.661	87	-2.105
**miR870**	14	15	0.326	21	0.612	6	-1.134
**miR1886**	52	70	0.655	75	0.556	85	0.797
**miR1888a**	74	55	-0.202	125	0.784	49	-0.506
**miR1888b**	48	44	0.101	52	0.143	58	0.361
**miR2111a**	2	67	5.292	9	2.197	28	3.896
**miR2111b**	3	47	4.196	8	1.442	43	3.930
**miR2112**	2	10	2.548	10	2.349	10	2.410
**miR2933a**	94	134	0.738	115	0.318	84	-0.074
**miR2933b**	70	108	0.852	84	0.290	63	-0.064
**miR2936**	32	39	0.512	52	0.728	43	0.515
**miR3434**	245	439	1.068	420	0.805	301	0.385
**miR3440b**	11	24	1.352	24	1.153	27	1.384
**miR3932a**	56	17	-1.494	48	-0.195	54	0.036
**miR3932b**	2516	110	-4.289	1408	-0.810	1153	-1.037
**miR4221**	12	33	1.686	12	0.027	7	-0.689
**miR4228**	82	178	1.344	77	-0.063	93	0.270
**miR4245**	475	452	0.155	537	0.204	424	-0.076

**miR5012**	37	85	1.426	136	1.905	89	1.355
**miR5014a**	63	17	-1.664	46	-0.426	16	-1.889
**miR5014b**	254	73	-1.573	222	-0.167	76	-1.652
**miR5020a**	35	4	-2.903	30	-0.195	30	-0.134
**miR5020b**	106	13	-2.801	37	-1.491	22	-2.180
**miR5024**	45	93	1.274	113	1.356	90	1.088
**miR5026**	4861	2392	-0.797	6738	0.498	22173	2.278
**miR5027**	10	5	-0.774	21	1.098	9	-0.064
**miR5028**	4	115	5.072	17	2.115	17	2.176
**miR5029**	8	13	0.927	10	0.349	11	0.548
**miR5595a**	171	290	0.988	231	0.461	202	0.329
**miR5628**	5	3	-0.511	25	2.349	7	0.574
**miR5629**	26	99	2.155	46	0.850	83	1.763
**miR5630b**	11	1	-3.233	2	-2.432	2	-2.371
**miR5632**	7	20	1.741	6	-0.195	27	2.036
**miR5633**	10	14	0.712	16	0.705	14	0.574
**miR5635a**	592	492	-0.041	697	0.263	308	-0.854
**miR5635b**	411	249	-0.497	354	-0.188	218	-0.826
**miR5635c**	203	164	-0.082	237	0.251	141	-0.437
**miR5635d**	394	312	-0.110	439	0.183	254	-0.545
**miR5636**	7	5	-0.259	11	0.679	4	-0.719
**miR5637**	28	76	1.667	122	2.151	130	2.303
**miR5638a**	13	12	0.111	38	1.575	14	0.195
**miR5638b**	4	8	1.226	11	1.487	7	0.896
**miR5639**	6	13	1.342	6	0.027	3	-0.912
**miR5640**	64	60	0.133	99	0.657	48	-0.327
**miR5641**	21	20	0.156	47	1.190	41	1.054
**miR5642a**	3830	4683	0.516	4376	0.220	5711	0.665
**miR5642b**	961	1383	0.751	1467	0.638	2233	1.305
**miR5643a**	166	161	0.182	145	-0.168	95	-0.717
**miR5643b**	153	74	-0.822	93	-0.691	47	-1.614
**miR5644**	696	237	-1.328	481	-0.506	267	-1.294
**miR5645a**	461	234	-0.752	418	-0.114	214	-1.019
**miR5645b**	405	189	-0.873	318	-0.322	164	-1.216
**miR5645c**	73	61	-0.033	52	-0.462	69	0.007
**miR5645d**	243	136	-0.611	191	-0.320	113	-1.016
**miR5645e**	90	86	0.161	93	0.075	62	-0.449
**miR5645f**	315	188	-0.518	301	-0.038	164	-0.853
**miR5647**	35	99	1.726	59	0.781	64	0.959
**miR5650**	127	41	-1.405	149	0.258	90	-0.408
**miR5651**	91	97	0.318	200	1.163	94	0.135
**miR5652**	300	166	-0.628	735	1.320	420	0.574
**miR5653**	1219	1143	0.133	1599	0.419	911	-0.332

**miR5654**	292	363	0.540	693	1.274	334	0.282
**miR5655**	4	55	4.008	8	1.027	15	1.995
**miR5656**	26	26	0.226	51	0.999	44	0.847
**miR5657**	41	89	1.344	68	0.757	69	0.839
**miR5659**	89	34	-1.162	71	-0.299	37	-1.178
**miR5662**	492	183	-1.201	356	-0.439	196	-1.239
**miR5663**	87	138	0.892	178	1.060	156	0.931
**miR5664**	41	60	0.776	49	0.285	24	-0.684
**miR5665**	290	133	-0.898	266	-0.097	138	-0.983
**miR5995b**	55	54	0.200	32	-0.754	31	-0.739
**miR5996**	623	320	-0.735	491	-0.316	879	0.585
**miR5997**	6	2	-1.359	13	1.143	6	0.088
**miR8121**	66	99	0.811	91	0.491	60	-0.049
**miR8165**	72	39	-0.658	100	0.501	39	-0.796
**miR8166**	202	146	-0.242	224	0.177	112	-0.763
**miR8167a**	115	60	-0.712	118	0.065	64	-0.757
**miR8167b**	115	62	-0.665	113	0.002	65	-0.735
**miR8167c**	108	61	-0.598	114	0.105	72	-0.497
**miR8167d**	112	56	-0.774	116	0.078	74	-0.510
**miR8167e**	116	66	-0.587	119	0.064	76	-0.522
**miR8167f**	119	64	-0.669	118	0.015	63	-0.829
**miR8168**	34	52	0.839	54	0.695	22	-0.540
**miR8169**	278	101	-1.234	153	-0.834	76	-1.783
**miR8170**	24	12	-0.774	37	0.652	22	-0.037
**miR8171**	138	99	-0.253	106	-0.353	93	-0.481
**miR8172**	383	221	-0.567	372	-0.015	198	-0.864
**miR8173**	128	255	1.221	131	0.061	96	-0.327
**miR8174**	112	48	-0.996	75	-0.551	31	-1.765
**miR8175**	173	133	-0.153	211	0.314	217	0.415
**miR8176**	41	63	0.846	44	0.129	81	1.071
**miR8177**	99	42	-1.011	89	-0.126	31	-1.587
**miR8178**	97	68	-0.286	62	-0.618	67	-0.445
**miR8179**	7	8	0.419	14	1.027	12	0.866
**miR8180**	285	303	0.315	311	0.153	179	-0.583
**miR8182**	6	6	0.226	14	1.250	10	0.825
**miR8183**	18	37	1.266	42	1.250	20	0.240
**Library Size**	**30,635,847**	**26,189,325**		**30,060,261**		**28,816,378**	
**Number of miRNA reads**	*463,695*	*604,486*		*1,101,538*		*750,173*	
**% of Library**	***1.5***	***2.3***		***3.7***		***2.6***	

Profiling the abiotic stress responsive microRNA landscape of *Arabidopsis thaliana*

Joseph L Pegler[1], Jackson MJ Oultram[1], Christopher PL Grof[1] and Andrew L Eamens[1,*]

[1] Centre for Plant Science, School of Environmental and Life Sciences, Faculty of Science, University of Newcastle, Callaghan 2308, New South Wales, Australia

* Correspondence: E-mail: andy.eamens@newcastle.edu.au; Tel.: +61-249-217-784

Supplemental Table S2: Sequences of the DNA oligonucleotides used in this study for the synthesis of miRNA-specific cDNAs and the RT-qPCR based quantification of miRNA abundance or miRNA target gene expression.

The DNA oligonucleotide sequence of the stem-loop primer used to prime the reverse transcription of a miRNA-specific cDNA for each miRNA sRNA of interest is provided in the below Table (denoted by RTSL). Post synthesis of a miRNA-specific cDNA, the miRNA-specific forward primer (denoted by RTF in the below Table) and a generic reverse primer (denoted by SLR in the below table (underlined sequence of each RTSL primer identifies the binding site for the generic reverse primer)) were used to quantify the abundance of each miRNA sRNA. Also provided in the below Table is the sequence of the DNA oligonucleotide used as the forward (denoted by RTF in the below Table) and reverse (denoted by RTR in the below Table) primer to quantify the expression level of a target gene of each miRNA of interest. The snoRNA, *snoR101*, was used to normalize the abundance value of each miRNA sRNA across the non-stressed control and heat, drought and salt stressed samples. The reference gene, *UBI10* (*AT4G05320*), was used to normalise the expression of each miRNA target gene assessed across the non-stressed control and heat, drought and salt stressed *Arabidopsis* whole seedlings samples.

Targeted Sequence	Primer Name	Oligonucleotide sequence (5' to 3')
**DNA oligonucleotides used for miRNA-specific cDNA synthesis and RT-qPCR abundance quantification**		
**miR156**	**p156-RTSL**	GTCGTATCCAGTGCAGGGTCCGAGGTATTCGCACTGGATACGACGTGCTC
	**p156-RTF**	CGCCTGACAGAAGAGAGTGAGCAC
**miR169**	**p169-RTSL**	GTCGTATCCAGTGCAGGGTCCGAGGTATTCGCACTGGATACGACCGGCAA
	**p169-RTF**	AGTGAGCCAAGGATGACTTGCCG
**miR395**	**p395-RTSL**	GTCGTATCCAGTGCAGGGTCCGAGGTATTCGCACTGGATACGACGAGTTC
	**p395-RTF**	GCGCTGAAGTGTTTGGGGGAACTC
**miR396**	**p396-RTSL**	GTCGTATCCAGTGCAGGGTCCGAGGTATTCGCACTGGATACGACAAGTTC
	**p396-RTF**	GCGCGTTCCACAGCTTTCTTGAAC
**miR399**	**p399-RTSL**	GTCGTATCCAGTGCAGGGTCCGAGGTATTCGCACTGGATACGACCAGGGC
	**p399-RTF**	GCATGCCAAAGGAGATTTGCCCTG
**miR778**	**p778-RTSL**	GTCGTATCCAGTGCAGGGTCCGAGGTATTCGCACTGGATACGACCGGTGT
	**p778-RTF**	GGCGTGGCTTGGTTTATGTACACCG
**miR839**	**p839-RTSL**	GTCGTATCCAGTGCAGGGTCCGAGGTATTCGCACTGGATACGACGGGAAC
	**p839-RTF**	AGCGTACCAACCTTTCATCGTTCCC
**miR855**	**p855-RTSL**	GTCGTATCCAGTGCAGGGTCCGAGGTATTCGCACTGGATACGACTTCCTT
	**p855-RTF**	GGCGGAGCAAAAGCTAAGGAAAAGG
**miR857**	**p857-RTSL**	GTCGTATCCAGTGCAGGGTCCGAGGTATTCGCACTGGATACGACATACAC
	**p857-RTF**	GGCGGCGTTTTGTATGTTGAAGGTG
**miRNA stem-loop oligo**	**pSLR-Generic**	CCAGTGCAGGGTCCGAGGTA
**snoR101**	**pSnoR-RTF**	CTTCACAGGTAAGTTCGCTTG
	**pSnoR-RTR**	AGCATCAGCAGACCAGTAGTT

**DNA oligonucleotides used for miRNA target gene expression quantification via RT-qPCR**		
***ATPS1* (*AT3G22890*)**	**pATPS1-RTF**	ATCTCCGGCACTAAGATGCG
	**pATPS1-RTR**	ACCTGGGCACATAAAACCGT
***GRF7* (*AT5G53660*)**	**pGRF7-RTF**	CATCCCCCACCGTTAGATCG
	**pGRF7-RTR**	TGCTTCCATGCTTCCGACAT
***LAC7* (*AT3G09220*)**	**pLAC7-RTF**	ACACACCTTCAACGTACAAAACT
	**pLAC7-RTR**	ACCCTCCTTGACGCGTATTG
***NFYA5* (*AT1G54160*)**	**pNFYA5-RTF**	ACCAAATCCAAAGCACCAAAGT
	**pNFYA5-RTR**	AGGCATTGAGTTTCCCCAAGA
***P5CS1* (*AT2G39800*)**	**pP5CS1-RTF**	GTTTTTGAATCCCGACCTGA
	**pP5CS1-RTR**	TTACCCCCAACAGTCTCTGG
***PHO2* (*AT2G33770*)**	**pPHO2-RTF**	ACCGTTTCTCATCAAGGCGT
	**pPHO2-RTR**	GTGCCCGTCCACCATAAGAA
***SPL9* (*AT2G42200*)**	**pSPL9-RTF**	TTTTGGCCCGATGACGGTTA
	**pSPL9-RTR**	AATACCCAAGGCGGGTTCAG
***SUVH6* (*AT2G22740*)**	**pSUVH6-RTF**	TTGCAGTTGCAAAACCGAGG
	**pSUVH6-RTR**	TCCTTCACCAAACTCTCGGC
***UBI10* (*AT4G05320*)**	**pUBI10-RTF**	GGCCTTGTATAATCCCTGATGAATAAG
	**pUBI10-RTR**	AAAGAGATAACAGGAACGGAAACATA
